# The evaluation of right and left ventricular morphology by CMR with comparison to recipient heart after heart transplant: a surgical perspective

**DOI:** 10.1186/1532-429X-11-S1-P36

**Published:** 2009-01-28

**Authors:** Nicholas Farber, Mark Doyle, Ronald B Williams, June A Yamrozik, Perry Bruno, George J Magovern, James A Magovern, Srinivas Murali, Robert WW Biederman

**Affiliations:** Allegheny General Hospital, The Gerald McGinnis Cardiovascular Institute, Pittsburgh, PA USA

**Keywords:** Interventricular Septum, Steady State Free Precession, Cardiac Mass, Donor Heart, Initial Validation

## Introduction

Cardiovascular MRI (CMR) is considered the "gold standard" for non-invasive left and right ventricular mass quantitation. To our knowledge, this information is only based on animal and phantom data and has never been prospectively or retrospectively validated in humans undermining the credibility of the 'gold standard'. This issue is particularly important for the right ventricle having complex geometry ill suited for mathematical modeling, placing increased importance on accurate mass quantitation. The surest way to validate the accuracy and thus the *true* gold standard of CMR derived mass is through autopsy.

## Purpose

To establish a correlation between CMR derived ventricular mass and autopsy mass of *ex vivo* hearts from heart transplants.

## Methods

Over a 10-week period, five *ex vivo* hearts donor hearts were obtained immediately upon orthotopic heart transplantation from the operating room. They were quickly cleaned and suspended in a saline-filled container and scanned using a steady state free precession (SSFP) sequence and 2D short-axis slices to measure the CMR defined weight (g), (GE 1.5 T, Excite. Milwaukee, WI). Semiautomated endocardial and epicardial contouring was performed via Mass Plus, Medis (The Leiden, The Netherlands). The donor hearts were then dissected to shave the atria off at the atrioventricular plane and ventricles separated at the interventricular septum. The actual weight of the LV and RV was measured via high-fidelity scale giving the true mass of each ventricle for comparison with the weighed mass.

## Results

The CMR measured LV mass (mean = 329.2 g, SD = 78.9 g) significantly predicted the actual measured LV mass (mean = 329.4 g, SD = 83.7 g), p = NS. The Pearson product-moment correlation for this sample was 0.94 (p = 0.02). The CMR measured RV mass (mean = 151.0 g , SD = 50.5 g) significantly predicted the actual measured RV mass (mean = 126.8 g, SD = 44.6 g). The Pearson product-moment correlation for this sample was 0.92 (p = 0.03). The CMR measured LV + RV mass (mean = 240.1 g, SD = 112.8 g) significantly predicted the actual measured LV + RV mass (mean = 228.1 g, SD = 124.1 g). The Pearson product-moment correlation for this sample was 0.98 (p < 0.001).

## Conclusion

CMR accurately determined both left and right ventricular masses as compared to weighed explanted hearts, despite variable surgical removal of instrumentation (LVAD/RVAD and AICD's) in the majority and the complexities of the right ventricle in all. To our knowledge, albeit a small sample size, it represents a 'first' in human CMR vs. autopsy comparison, similar to the intrepid days of the initial validation of echocardiography vs. autopsy report by Reichek and Devereux in 1976. Future work will include sampling of additional *ex vivo* hearts and a correlation between *in vivo* CMR derived cardiac mass pre-heart transplant and following explantation.

